# Animal Toxins Providing Insights into TRPV1 Activation Mechanism

**DOI:** 10.3390/toxins9100326

**Published:** 2017-10-16

**Authors:** Matan Geron, Adina Hazan, Avi Priel

**Affiliations:** The Institute for Drug Research (IDR), School of Pharmacy, Faculty of Medicine, The Hebrew University of Jerusalem, Jerusalem 9112001, Israel; matan.geron@mail.huji.ac.il (M.G.); adina.hazan@mail.huji.ac.il (A.H.)

**Keywords:** TRPV1, outer pore domain, spider toxin, centipede toxin, scorpion toxin, snake toxin, sea anemone, nociception, venom, pain

## Abstract

Beyond providing evolutionary advantages, venoms offer unique research tools, as they were developed to target functionally important proteins and pathways. As a key pain receptor in the nociceptive pathway, transient receptor potential vanilloid 1 (TRPV1) of the TRP superfamily has been shown to be a target for several toxins, as a way of producing pain to deter predators. Importantly, TRPV1 is involved in thermoregulation, inflammation, and acute nociception. As such, toxins provide tools to understand TRPV1 activation and modulation, a critical step in advancing pain research and the development of novel analgesics. Indeed, the phytotoxin capsaicin, which is the spicy chemical in chili peppers, was invaluable in the original cloning and characterization of TRPV1. The unique properties of each subsequently characterized toxin have continued to advance our understanding of functional, structural, and biophysical characteristics of TRPV1. By building on previous reviews, this work aims to provide a comprehensive summary of the advancements made in TRPV1 research in recent years by employing animal toxins, in particular DkTx, RhTx, BmP01, *Echis coloratus* toxins, APHCs and HCRG21. We examine each toxin’s functional aspects, behavioral effects, and structural features, all of which have contributed to our current knowledge of TRPV1. We additionally discuss the key features of TRPV1’s outer pore domain, which proves to be the target of the currently discussed toxins.

## 1. Background

Noxious stimuli are detected by a wide array of peripherally located ion channel receptors, where their activation is transferred to the central nervous system mainly via small diameter, unmyelinated c fibers [1]. Due to the limited number of receptors charged with detecting innumerable stimuli, many of the ion channel receptors have the ability to detect and respond appropriately to a multitude of stimuli (polymodality) [2,3]. One such receptor, TRPV1, has the ability to detect internal and external noxious stimuli such as high temperature (>42 °C), low pH, peptide toxins and capsaicin, the “hot” chemical in chili pepper [4,5,6]. TRPV1 activation, sensitization, and modulation have been indicated in many diseases, including irritable bowel syndrome, cancer, and diabetes [7,8,9,10].

**Figure 1:**
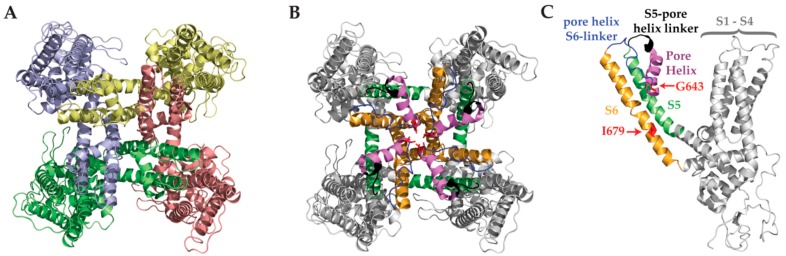
Pore-forming domains in the TRPV1 tetramer. (**A**) Top view of closed tetrameric TRPV1 channel showing transmembrane helices and emphasizing the intertwined subunits arranged around a central pore. Each subunit is color-coded individually. PDB ID: 5IRZ. (**B**) Color coded outer pore domain and pore-forming structures from a top-down view of the tetrameric channel. S5 (green) links with the pore helix (pink) via its linker (black). The pore helix connects to S6 (gold) via an outer pore linker (blue), which also harbors the upper selectivity filter Gly 643 (red). A lower selectivity filter (red) appears further down S6 at Ile 679 (red). S1–S4 are represented in grey. Note that the pore turret (23 AA) situated between S5 and the pore helix is omitted in this structure. PDB ID: 5IRZ. (**C**) Side view of a single TRPV1 subunit color coded as described in B. PDB ID: 5IRZ.

Structurally, the TRPV1 receptor consists of four independent, identical protein subunits that assemble into a functional, non-selective cation channel (Figure 1) [11]. Each of the four subunits is composed of six transmembrane segments, with a pore-forming loop between segments five and six (pore helix) and intracellular *N*- and *C*- termini (Figure 1) [11,12]. Importantly, 25 amino acids in the S5 outer pore domain leading to the pore helix make up the pore turret, which has been proposed to be involved in heat-induced activation [13]. Over 112 known functional sites found along the sequence of each subunit are responsible for TRPV1’s capacity to respond to a multitude of agonists, antagonists, and channel blockers [14]. One major binding site is the S3–S4 located vanilloid binding site, which is activated by endo-vanilloids such as anandamide, or exo-vanillolids such as capsaicin [15,16,17,18]. A second major binding domain has been described as the outer pore region, essential for proton-mediated TRPV1 activation [5]. Following the successfully constructed cryo-EM structure, we now understand that TRPV1 boasts a relatively broad outer pore domain, which has been suggested to enhance accessibility to ligands of variable size and charge [19]. Indeed, to date, all characterized animal toxins’ binding sites were found to reside in the outer pore domain. During TRPV1 gating, considerable rearrangements occur in the outer pore, pore helix, and selectivity filter, but not in transmembrane segments 1–4 as would be expected when compared to the structurally-similar voltage gated channels [11,12,20]. In addition, TRPV1 activation is distinct in that it depends on the opening of two allosterically coupled gates. Large structural rearrangements in the outer pore domain affect the pore helix and selectivity filter, while a hydrophobic narrowing of the lower gate undergoes expansion during activation [11]. Functionally, it has been shown that activation of single subunit through the vanilloid binding site is sufficient to activate the entire channel [21]. Activation via the outer pore domain by protons requires four functional binding sites, indicating a discrepancy in the coupling mechanism between the two gates and the opening of the pore [21]. 

Due to its major role in the pain pathway, TRPV1 offers an attractive target for pharmacological manipulation in pain management [22]. There have been many attempts to design functional TRPV1 antagonists, which could potentially block pain transmission from the periphery. Even prior to the cloning of TRPV1, the potent and specific antagonist capsazepine was synthesized by modifying the structure of the naturally-occurring plant toxins, capsaicin and resiniferatoxin, in order to block nociceptive firing and potentially be used as an analgesic [23]. However, this molecule, along with many other molecules synthesized since then, has been found unsuitable for therapeutic use. The major stumbling blocks in successful pain therapy through TRPV1 inhibition are critical side effects such as hyperthermia and changes in core body temperature, or decreased noxious heat detection due to TRPV1’s role in thermo-regulation and sensation [24]. A more successful approach, albeit imperfect, has been to take advantage of TRPV1 desensitization that occurs during strong activation by potent agonists such as capsaicin [25]. This approach is currently in use as a topical analgesic cream, but maintains side effects such as an initial burning sensation and potential damage to nociceptors [26]. Accordingly, more research is necessary to understand the function and regulation of TRPV1 in pain in order to develop molecules which affect only a single modality of the channel instead of blocking the channel’s full array of functions.

Overall, an organism’s nociceptive system serves as a beneficial protective mechanism to prevent or respond to tissue damage [2]. Venomous animals take advantage of this system and have developed highly specific, potent, and complex toxins to specifically target sensitive proteins in the pain system, such as TRPV1 [6]. As such, these molecules provide unique probes for understanding functionally important proteins in the peripheral pain pathway.

TRPV1’s original cloning and characterization was made possible by the plant toxin capsaicin, used as a probe to search for a heat channel in the pain pathway [4]. Since then, capsaicin has become the “gold standard” of TRPV1 activation, and has contributed to critical developments over the last 20 years in understanding the role of TRPV1 in pruritis, cancer, weight loss, and in the cannabinoid system [8,9,27,28]. Resiniferatoxin (RTX), an ultra-potent phytotoxin agonist that is found in the plants *Euphorbia resinifera* and *Einhorbia poissonii* found in Morocco, has likewise become an invaluable research tool [29,30]. The successful elucidation of the TRPV1 cryo-EM structure in the open state was possible due to the extreme affinity and binding of RTX paired with the irreversible outer-pore binding Double knot Toxin (DkTx) [11]. Functionally, due to the strong activation and high affinity binding to TRPV1, RTX has been shown to selectively ablate TRPV1^+^ nociceptors. Taking advantage of this, RTX administration is being clinically investigated as an analgesic in several ailments, such as severe burn subjects or bone cancer [31]. Additionally, vanillotoxins (VaTx1, VaTx2 and VaTx3), from the venom of the tarantula *Psalmopoeus cambridgei*, were the first described animal derived peptides that activate the somatosensory system, in this case specifically targeting TRPV1 (previously reviewed) [6,32]. This set of toxins, similar in structure to each other, contains three inhibitory cysteine knot (ICK) peptides [32]. ICK is a structural motif shared by toxins from venomous animals of many species, such as cone snails, spiders and scorpions [32]. These toxins have a unique feature of six cysteine residues that form sulfide bridges, producing a “knot-like” structure [33]. ICK toxins are most commonly involved in channel inhibition, such as Kv channels, causing paralysis and hyperexcitability [33]. Finally, some venomous animals (e.g, funnel web spider and *Heteractis crispa* sea anemone) were shown to produce toxins that inhibit TRPV1 rather than activating it. The purpose of these presumably analgesic toxins remain unknown [34].

Overall, toxins have provided unparalleled tools in understanding TRPV1 activation, regulation, and structure. In the pursuit to design more efficient and specific pain treatments through TRPV1 modulation, we further turn to toxins to elucidate the different binding sites of TRPV1 and its activation mechanism(s). To date, several animal toxins have been described that activate, modulate, or inhibit TRPV1. In this review, we focus on the recent animal toxins research that have contributed to our understanding of this receptor’s unique activation profile and its inner workings as a receptor in the pain pathway.

## 2. Double-Knot Toxin (DkTx)

### 2.1. Introduction

**Figure 2:**
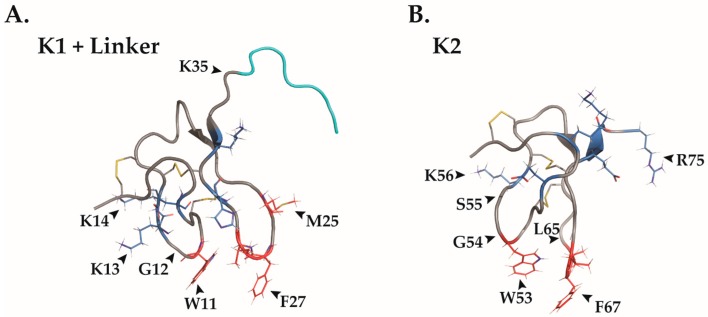
The amphipathic nature of the DkTx structure allows it to protrude the membrane bilayer. Individually visualized knots of DkTx, with the linker (cyan) between the two knots appearing on K1 (**A**) Hydrophobic residues are labeled in red and polar residues in blue. This amphipathic nature presumably enables DkTx to successfully protrude into the lipid environment of the cell membrane. Key amino acids indicated in TRPV1 binding according to computational scan studies have been labeled (). Disulfide bridges are labeled in yellow. K1, PDB ID2N9Z. (**B**) K2 knot of DkTx labeled as described in A. Amino acids in K2 are numbered according to the molecule in its entirety.PDB ID: 2NAJ.

Vanillotoxins are ICK motif-containing spider proteins targeting TRPV1 [6,32]. These toxins activate the channel by binding to the extracellular pore domain [35]. One of these vanillotoxins, DkTx (8522 Da), has a unique bivalent structure: two tandemly repeated ICK motifs that are highly homologous (67% identity, Figure 2) [36,37]. This TRPV1-selective toxin is found in the venom of the Chinese bird spider (*Ornithoctonus huwena*), an old world aggressive tarantula [36,38]. Its bivalent structure allows DkTx to form an exceptionally stable complex with TRPV1’s outer pore region resulting in persistent current conductance through the channel [36,37,39,40]. In contrast, the separated DkTx ICKs (knot 1, “K1”; knot 2, “K2”) as well as the single knot vanillotoxins, VaTx1-3, produce reversible binding and activation of TRPV1 [36,39]. Additionally, DkTx’s bivalency accounts for its increased potency and avidity of TRPV1 activation in comparison to other vanillotoxins [36,41].

### 2.2. Functional Aspects

An interesting feature of DkTx-induced TRPV1 current is its relatively slow kinetics during activation until maximal amplitude is reached [36]. However, co-application of DkTx and capsaicin results in increased binding and activation rates for the toxin as evident by faster onset of a stable, irreversible current typical to DkTx on both the single channel and whole cell levels [36]. Thus, it was suggested that DkTx preferentially binds, and subsequently locks, TRPV1 in the channel’s open conformation [20,36,42]. Accordingly, in the absence of another activating stimulus, DkTx presumably binds TRPV1 during one of the channel’s brief spontaneous transitions to the open state [20]. Further support for this notion can be derived from the apo structure of TRPV1 in which superimposition of DkTx clashes with the channel’s side chains [20]. Thus, it is possible that channel activation by DkTx in low temperatures, when the open state excursions of TRPV1 are diminished, could be reduced. In addition, it could be expected that the toxin’s ability to irreversibly lock and stabilize TRPV1 in its open state would yield maximal open probability, especially when compared to the flickering nature of channel activation by the reversible agonist capsaicin [36,43,44].

### 2.3. Effect on Nociception

Considering the toxin’s unique activation profile of TRPV1, DkTx is expected to cause an excruciating and prolonged pain response [36]. Indeed, the bite of a Chinese-bird spider reportedly produces substantial pain and inflammation [38]. Nonetheless, experiments evaluating the specific effects of DkTx on pain and aversive responses have yet to be conducted. Additionally, the neuronal firing properties of TRPV1-positive nociceptors in response to DkTx application has yet to be characterized. Thus, whether prolonged TRPV1 activation by the toxin indeed leads to prolonged action potential firing on the neuronal level is still unknown.

### 2.4. Structural Features

A recent cryo-EM structure in near-atomic resolution of the DkTx-TRPV1 complex revealed that the two toxins’ ICK motifs bind two adjacent subunits in the homo-tetrameric channel in an anti-parallel orientation [11,20,45]. Therefore, two DkTx molecules can fully occupy a single TRPV1 channel by binding to its outer pore domain [20,39,46]. DkTx’s linker consists of seven amino acids, including two proline residues, which likely reduce its flexibility [36,39]. In addition, the toxin’s linker adopts a tense and constrained conformation upon DkTx binding to TRPV1, which may reflect a linker’s length that has evolved to match the distance between two adjacent ICK binding sites [20]. As different activators display a distinct activation stoichiometry of TRPV1, it is still not known how many knots bound to the channel are required in order to elicit channel gating [21]. In each TRPV1 subunit, the different toxin knots bind equivalent binding sites that are situated in the interface of two neighboring subunits [11,39]. Thus, when bound, a single knot interacts with the pore helix of one subunit and the pore loop preceding S6 in an adjacent subunit [20]. A previous alanine scan study has identified four TRPV1 residues in the S5-pore helix loop (I599; rTRPV1), S6-pore helix loop (F649; rTRPV1) and S6 (A657 and F659; rTRPV1) which are critical to channel activation by DkTx [36]. Additionally, molecular-dynamics simulations analysis suggested several TRPV1 residues that are in proximity to the bound DkTx and may be involved in toxin binding [39]. These include K535 and E536 (S4) (rTRPV1), Y631 (pore helix) (rTRPV1) and a stretch of residues between the pore helix and S6 (F649, T650, N652, D654, F655, K656, A657 and V658; rTRPV1). Thus, it was postulated that the more distant I599 and F659 (rTRPV1) contribute to DkTx-induced gating but not directly to the binding of the toxin [39]. It was further suggested that these two residues along with V595, F649 and T650 (rTRPV1) form a hydrophobic cluster which lies in the interface between the S5, S6, and the pore helix, and behind the selectivity filter [39]. Following DkTx binding, it is presumed that this cluster is disrupted and that residues in the cluster undergo substantial conformational changes which lead to ion permeation through the channel [39].

Although DkTx’s ICK motifs are highly homologous and share the same binding site, they differ in binding orientation, leading to a higher potency observed in K2 than K1 [36,39]. Computational alanine scanning revealed that most residues involved in channel binding are conserved between the two ICKs as W11, G12, K14 and F27 from K1 as well as W53, G54, K56 and F67 from K2 were found to play an important role in TRPV1 activation (Figure 2) [39]. However, functional differences between the two knots could stem from other residues crucial for toxin binding which are variable between K1 and K2 such as K13 and S55, M25 and L65, and K35 and R75, respectively (Figure 2) [39]. Interestingly, these alternate residues are situated in a region that was shown to influence the knot’s affinity to TRPV1 in a chimera study [39]. DkTx contains a large hydrophobic surface as each ICK contains two hydrophobic fingertips (K1: W11, M25, F27, and I28; K2: W53, L65, A66, F67, and I68) (Figure 2). However, these fingers are surrounded by acidic and basic residues (K1: E7, K13, K14, H30, and K32; K2: E47, E49, K56, E72, K73, and R75) (Figure 2) [20,39]. Similar amphipathic nature was previously shown to allow other ICK toxins to protrude into lipid environments [47,48,49,50]. Furthermore, tryptophan (Trp) fluorescence showed that DkTx is indeed able to undergo partitioning into lipid environments [39]. In addition, it was found that K1 permeates the membrane more efficiently than K2 [39]. Thus, it was speculated that in the course of DkTx binding, K1 initially partitions the membrane and then coordinates the binding of its more potent counterpart, K2, to TRPV1 [39]. However, there is still no evidence that this is indeed the case. An improved-resolution cryo-EM structure of the DkTx-bound TRPV1 shows that DkTx hydrophobic fingers penetrate 9 Å deep into the membrane thus causing local distortions in the lipid bilayer [20]. Moreover, this structure revealed that some membrane lipids form interactions with both DkTx fingers and TRPV1 residues thus producing a tripartite complex. For instance, DkTx’s W11 (K1) and W53 (K2) form hydrophobic interactions with aliphatic chains whose polar head groups bind to R534 (S4; rTRPV1) in TRPV1 [20]. Other examples are F27 (K1) and F67 (K2) that similarly interact with triglycerides which are also in contact with S629 (pore helix; rTRPV1) as well as Y453 (S1; rTRPV1) [20]. These toxin-lipid-channel interactions along with DkTx-TRPV1 bonds likely compensate for the energetic penalty predicted for the interference of the organized lipid environment in this structure [20,39]. Overall, the interaction surface between DkTx and TRPV1 is quite limited as the bound toxin is also stabilized via its interaction with the lipid bilayer [20].

## 3. RhTx

### 3.1. Introduction

RhTx is a peptide toxin (27 amino acids) that was identified in the venom of the Chinese red-headed centipede (*Scolopendra subspinipes mutilans*) [51]. This aggressive arthropod, which populates parts of eastern Asia and Australasia, can cause extreme localized pain upon envenomation [51,52]. It was found that RhTx is a selective TRPV1 activator, as it does not affect other TRPV channels (i.e., TRPV2-4). In addition, the voltage-gated potassium ion channel Kv2.1, which was previously shown to be inhibited by other peptide toxins targeting TRPV1 (i.e., VaTx1 and VaTx2), is neither inhibited nor activated by RhTx [32,51]. Similar to capsaicin-mediated TRPV1 activation, RhTx displays very rapid kinetics, in both channel opening and washout. This is in contrast to the slow-developing, slow washing DkTx described above [36,51].

### 3.2. Functional Aspects

RhTx activates TRPV1 with a comparable efficacy to capsaicin [51]. However, functional examinations revealed that TRPV1 activation by RhTx is highly temperature-dependent, as increased temperatures strongly potentiate the toxin activity [51,53]. In contrast to capsaicin, RhTx activity is significantly reduced at 20 °C compared to room temperature (RT) experiments and completely abolished at 10 °C [51]. Furthermore, 100 nM RhTx, 20% of the toxin’s EC_50_ at RT, reduces heat activation threshold below body temperatures [51]. Thus, RhTx is a potent TRPV1 activator in mammals’ physiological body temperatures. Single TRPV1 channel recordings revealed that RhTx evokes a near unity open probability while reduced current conductance was observed in negative holding membrane potentials in comparison to capsaicin [51]. Hence, additional experiments at negative holding potentials are required to understand the physiological relevance of the temperature-dependent activation by RhTx. In addition, RhTx desensitizes TRPV1’s response to heat, whereas activation by capsaicin is unaffected. These findings imply that RhTx affects the heat activation machinery of the channel either directly or allosterically [51]. However, the changes in toxin potency observed could merely be the result of preferential RhTx binding to the open state of TRPV1. Thus, further evidence is required to establish the functional interaction between RhTx and heat, as well as with other TRPV1 activators such as protons and capsaicin.

### 3.3. Effect on Nociception

RhTx was shown to evoke an acute pain response when injected into mice [51]. However, longer term possible implications of RhTx application such as sensitivity to heat, cold and tactile stimuli were not tested. Firing properties and desensitization of TRPV1 positive neurons activated by this toxin are yet to be characterized as well. In addition, while other potent toxins (e.g., capsaicin) targeting TRPV1 eventually cause TRPV1^+^ fiber denervation, it is still not known whether this is the case for RhTx. The RhTx-induced pain response observed was comparable to the response elicited by the crude centipede venom [51]. Thus, RhTx could account for the excruciating pain evoked by this centipede’s bite. Interestingly, a previously described toxin from the same venom, Ssm6a, was shown to selectively inhibit human and rodents Nav1.7 channels, causing analgesia [54]. Thus, the algogenic effect of this centipede’s venom is surprising given the prominent role of Nav1.7 in nociception [55].

### 3.4. Structural Features

**Figure 3:**
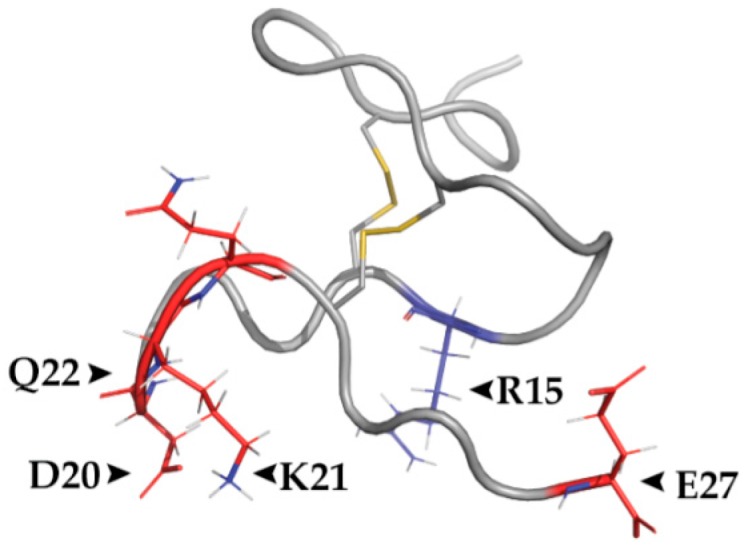
The polar RhTx binds TRPV1 through its charged surface. Structure of RhTx indicating the polarity of the molecule. Charged residues D20, K21, Q22 and E27 (red) have been indicated in TRPV1 binding, along with the polar residue R15. Two cysteine bridges are highlighted in yellow. PDB ID: 2MVA.

Structural analysis of RhTx revealed two disulfide bonds and a cluster of charged residues on one side of the molecule, endowing the toxin with polarity (Figure 3) [51,53]. Importantly, four charged residues (D20, K21, Q22 and E27) and one polar residue (R15) from the same structural domain were found to take part in RhTx-TRPV1 binding in a mutagenesis study, causing reduced or enhanced activation, respectively (Figure 3) [51]. These findings indicate that the toxin’s charged surface forms electrostatic interactions with the channel [51]. No effect on RhTx activity was detected when hydrophobic residues were mutated, implying there is no significant interaction between the toxin and the lipid bilayer [51]. Further analysis of channel interaction with RhTx identified D602 (mTRPV1 turret), Y632 and T634 (mTRPV1 pore helix) as critical to TRPV1 activation by this toxin. Using Rosetta-based molecular docking, RhTx was predicted to bind at the interface of two TRPV1 subunits similar to DkTx [20,51]. Thus, it is possible that the binding sites of these two toxins are overlapping. Interestingly, another TRPV1 residue (L461 of the mouse TRPV1) located within the S1–S2 extracellular linker was found to facilitate RhTx-induced TRPV1 activation. This observation points to a possible role of this region, which was previously thought to be stationary, in the activation mechanism of TRPV1 [51,56].

## 4. BmP01

### 4.1. Introduction

**Figure 4:**
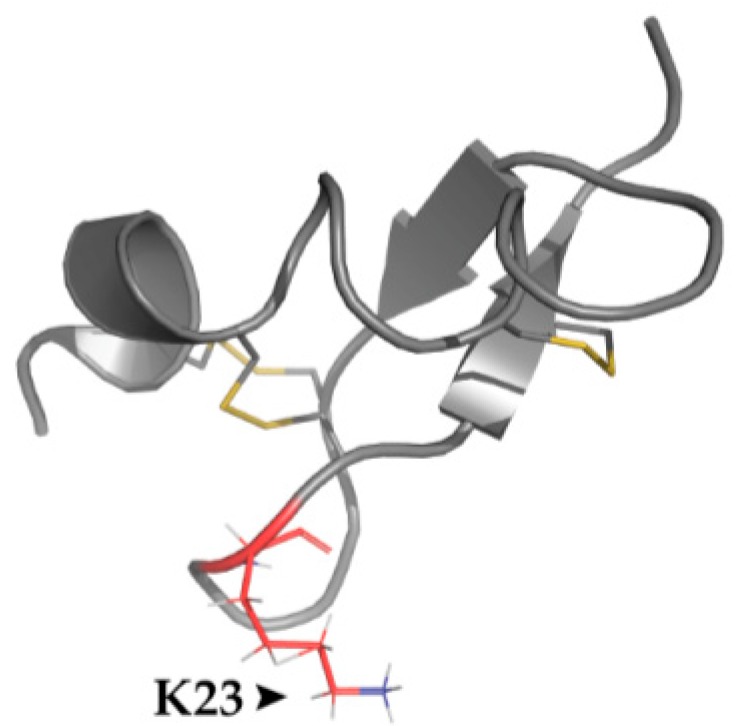
BmP01’s K23 forms an electrostatic interaction with TRPV1 outer pore region. Structure of BmP01 indicating the typical ICK motif formed by three disulfide bonds (indicated in yellow). Red indicates key amino acid K23, which interacts with E649 of TRPV1, an important proton-binding site in TRPV1 channel activation. PDB ID: 1WM7.

Scorpions are a source for diverse neurotoxins, which have contributed greatly to the study of their targeted ion channels [57]. BmP01, a 3178.6 Da (29 amino acids) protein, is the first scorpion toxin with TRPV1 activating properties to be described [58,59]. This toxin, found in the venom of *Mesobuthus martensii*, has a typical inhibitory cysteine knot (ICK) motif structure with three disulfide bonds (1–4, 2–5 and 3–6) stabilizing a compact and rigid protein fold (Figure 4) [58,59]. Within the vast ICK toxins family, BmP01 is further sub-classified as part of the a-KTX8 toxins subgroup with whom it shares similar topology and sequence features [60,61].

### 4.2. Functional Aspects

Concentration-response relationship experiments revealed that BmP01 dose-dependently activates the channel with comparable efficacy to capsaicin [59]. Accordingly, in single channel recordings it was shown that BmP01 produces conductance through TRPV1’s pore similar to capsaicin, albeit with reduced open probability [61]. The kinetics of BmP01 activity and washout in electrophysiological recordings from TRPV1 expressing cells also resembles capsaicin [61]. The desensitizing properties of BmP01 on TRPV1 were not tested so far. BmP01 does not activate TRPV3, a closely related channel to TRPV1 [61]. The effect of this toxin on other prominent pain sensing TRP channels (e.g., TRPA1, TRPM8, and TRPV2) has yet to be determined. However, in addition to TRPV1, BmP01 was also found to modulate the activity of voltage-gated potassium channels by potently inhibiting mKv1.3, hKv1.3, and rKv1.1, but not mKv1.1, thus presenting species specificity [59]. The bi-functionality of this toxin might enable an enhanced nociceptive response to BmP01 as both activating TRPV1 and inhibiting Kv channels would result in hyperexcitable nociceptors [32,61,62,63]. Remarkably, BmP01 displays strong pH-dependent activity [61]. BmP01 displays low potency at neutral pH (EC_50_ = 169.5 ± 12.3 µM) [59,61]. However, in acidic conditions the toxin’s inhibitory effect in Kv1.3 is diminished, whereas its agonistic effect on TRPV1 is greatly potentiated (EC_50_ = 3.76 ± 0.4 µM) [61]. This enhanced TRPV1 response is seen in pH values (~6.5) that were previously shown to sensitize the channel rather than activate it [5,61]. Thus, BmP01 and protons synergize to produce enhanced TRPV1 response [61]. Similar relations between BmP01 and heat (which similarly to protons modulates TRPV1 gating through the outer pore region), were yet to be reported. In addition, how BmP01 unique functional properties affect the neuronal response is yet to be characterized.

### 4.3. Effect on Nociception

Injection of 500 µM BmP01 evokes a pain response in wild-type mice but not in TRPV1 KO mice [59]. Therefore, this finding suggests that BmP01 does not activate other pain receptors in a physiologically significant manner. However, while BmP01-induced pain requires TRPV1, it is still not clear whether Kv1.3 contributes to this response. Nevertheless, it was shown that solely blocking this potassium channel does not evoke a pain response [59]. Furthermore, considering pH effect on both BmP01-dependent Kv inhibition and TRPV1 activation and that the pH of *Mesobuthus martensii* venom is acidic, TRPV1 is probably the main ion channel targeted by BmP01 to induce pain upon this scorpion’s sting [61]. Thus, the pH-dependent sensitization of TRPV1 towards BmP01 reflects a strategy which enables physiologically relevant toxin concentrations to inflict pain following scorpion envenomation [61]. Indeed, injecting BmP01 in an acidic solution potentiated the response to this toxin in mice while such an effect was absent for capsaicin in low pH solutions [61]. Increased sensitivity to heat and tactile stimuli were not tested following BmP01 application.

### 4.4. Structural Features

ICK motifs are a common structural feature among many neurotoxins including the TRPV1 targeting toxins, DkTx and VaTxs, which bind the outer pore domain of the tetrameric TRPV1 [36,64,65]. Indeed, chimera studies along with docking calculations indicated that BmP01 also binds this channel domain [61]. Site directed mutagenesis screening identified three polar residues (E649, T651, and E652; hTRPV1) involved in TRPV1 activation by BmP01 which are in proximity to residues implicated in vanillotoxins-induced gating [36,61]. Interestingly, these residues are situated in the pore helix-S6 loop that was shown to influence TRPV1 gating [56,61]. Specifically, E649 was shown to undergo protonation under acidic conditions that leads to channel activation [5]. Alanine scanning and thermodynamic cycle analysis revealed that BmP01’s K23 forms electrostatic interaction with E649 while the role of T651 and E652 as well as other possible interaction sites remain to be elucidated (Figure 4) [61]. Another site involved in proton-dependent TRPV1 gating is E601 (hTRPV1) which is protonated in slightly less acidic conditions than E649 [5]. Notwithstanding, while protonation at E601 only sensitizes TRPV1, protonation at both sites is required for robust channel activation [5,61]. Further functional tests showed that the protonation mimicking mutation, E601Q, potentiated BmP01 response in neutral pH while preventing protonation at this position by using the E601A mutation produced diminished toxin potency and pH dependence [61]. Thus, BmP01 takes advantage of the machinery mediating TRPV1 gating by protons to cause channel activation in pH values which otherwise only sensitize the channel [61].

## 5. *Echis coloratus* Toxins

### 5.1. Introduction

Pain is a hallmark of envenomation by most snakes [66]. However, little is known on how snake venoms produce this response, as only a few snake toxins targeting the pain pathway have been described so far [66,67,68]. Recently, peptides from the venom of the *Echis coloratus* viper were shown to activate TRPV1 [69]. This snake’s bite produces an intense local burning pain along with local swelling and hemorrhagic disturbances [70]. In a screening of RP-HPLC fractions of *Echis coloratus* venom, three (F1, F7, F13) out of 24 fractions were found to produce TRPV1 response [69]. However, the structural and functional properties of F1 and F7 remain unknown, as these fractions were not further analyzed. Moreover, while F13 was shown to contain several proteins, this fraction was not subjected to further purification steps and the TRPV1-activating entity was not isolated [69]. Thus, the structure of the toxin targeting TRPV1 was not determined. Nonetheless, denaturizing proteins in this fraction did not affect TRPV1 activation, indicating that the active compound is a low molecular weight peptide that presumably adopts a stable tight helical conformation similar to other heat resistant toxins previously described in snake venoms [69,71,72].

### 5.2. Functional Aspects

F13 evokes an outwardly rectifying TRPV1 current with extremely fast kinetics as F13 activation immediately terminates upon washout, similar to capsaicin and protons [69]. In addition, F13 evokes acute desensitization and tachyphylaxis of TRPV1 in the presence of calcium with typical kinetics [69]. F13 was shown to be a selective TRPV1 activator as no effect of this fraction was observed on other TRP channels expressed in nociceptors; TRPV2, TRPA1 and TRPM8 [69]. Modulation of voltage-gated ion channels by this fraction was not tested. In neurotropic assays, F13 presented NGF activity while SDS-PAGE analysis of this fraction revealed a protein with similar size to mouse NGF [69]. NGFs, which are commonly found in a variety of snake venoms, cause acute sensitization of TRPV1 indirectly through NGF receptors [73]. However, the TRPV1-activating component of this fraction was found to be independent of the NGF signaling pathway, as NGF receptor blockers do not alter TRPV1 response to F13 in a heterologous expression system [69]. These findings suggest that F13 contains both TRPV1 activating toxin and NGF, which likely synergize in vivo to produce an increased pain response mediated by TRPV1 [69].

### 5.3. Effect on Nociception

Envenomation by *Echis coloratus* snake results in hypotension, local swelling, necrosis and pain in humans [70,74,75]. While substances released from the damaged tissue are able to sensitize and activate nociceptors, this *Echis* snake was shown to produce also toxins that specifically target the pain pathway as reviewed here. However, as these TRPV1-targeting toxins were not isolated behavioral tests evaluating their specific effect *in vivo* are yet to be conducted.

### 5.4. Structural Features

The presumed peptide nature of the TRPV1-activating compound in F13 suggests that the binding site for this toxin resides in the extracellular domains of the channel. Indeed, mutations in the intracellular binding site for phytotoxins, the vanilloid binding site, that were previously shown to abolish capsaicin and resiniferatoxin activity do not influence F13-induced TRPV1 response [69]. Interestingly, the mutation A657P in the outer pore region of TRPV1, which renders the channel insensitive to DkTx and VaTx3, does not affect TRPV1 activation by F13 as well [69]. The possibility that F13 causes TRPV1 activation through the residues mediating protons-induced gating was not tested. Therefore, while this snake toxin likely binds the channel extracellularly, the exact binding site for the F13 toxin remains unknown. Additional mutagenesis and chimera studies along with structural and computational analysis of toxin structure and interactions with TRPV1 are required to locate this toxin’s binding site.

## 6. Analgesic Polypeptide *Heteractis crispa* (APHC) Toxins

### 6.1. Introduction

While we can logically understand that toxins have evolved to specifically activate the pain system, accumulating evidence suggests that many toxins have components that possess analgesic properties. Such examples include mambalgins from the Black mamba snake or the previously described non-peptide toxin from the spider *Agelenopsis aperta*, shown to inhibit ASICs and TRPV1, respectively [76,77]. The toxins APHC1-3, derived from the sea anemone *Heteractis crispa* found in the Indio–Pacific, were the first polypeptide inhibitor of TRPV1 described [78].

### 6.2. Functional Aspects

All three toxins were shown to be partial, but potent, antagonists, with APHC3 displaying the highest level of inhibition (71% of 3 µM capsaicin), and the lowest IC_50_ value (18 nM) [78]. As is common with molecules presenting with partial antagonistic characteristics, a bimodal mechanism of action of APHC on TRPV1 activity was recently described [79]. In the presence of saturating concentrations of capsaicin (3 µM), APHC acts as an inhibitor, whereas current produced by very low concentrations (3 nM) of capsaicin are potentiated in the presence of the toxin [79]. Interestingly, APHC1 showed the highest levels of potentiation, showing an increase of 250% of the capsaicin - evoked current [79]. Furthermore, APHC3 potentiated TRPV1 activation in response to slightly acidic (pH 6.2) solution, whereas no inhibition was observed at highly acidic (pH 4.5) solutions [79]. Likewise, low concentrations of the synthetic molecule 2-aminoethoxydiphenyl borate (2APB) were subject to APHC potentiation, while high concentrations were unaffected [79].

### 6.3. Effect on Nociception

In vivo tests suggest that APHC1 and APHC3 both have the ability to produce analgesia in different behavioral pain models [80]. Both toxins display a dose-dependent inhibition of thermal nociception in doses up to 0.1 mg/kg injected intramuscularly, intraperitoneally, and intravenously [80]. Interestingly, APHC1 is a more potent inhibitor, as doses as low as 0.01 mg/kg are effective in increasing paw withdrawal latency from a hot plate, while the minimum effective dose of APHC3 was 0.05 mg/kg [80]. In comparison, potent serine protease inhibitors with similar structure and folding had no effect on thermal sensation, indicating that the basic structure alone is not enough to affect TRPV1 mediated thermal sensation [80]. When the toxins were tested for their effects in the formalin test for inflammation, the two candidates produced different effects: whereas APHC1 decreased both phases of the formalin test (acute and inflammatory pain), APHC3 attenuated only the second phase, indicating the inhibition of TRPV1 was effective only in modulating TRPV1 during inflammation [80]. The proposed mechanism is that APHC3 inhibits pH mediated TRPV1 activation, found during the inflammatory response, whereas APHC1 modulates additional TRPV1 modalities [80]. On the other hand, both toxins attenuated thermal hyperalgesia observed during complete Freund’s adjuvant (CFA) injection in a dose dependent manner, although the effect of APHC1 was larger [80].

Similar to most molecules proposed to affect nociception through TRPV1, these molecules affect core body temperature [80]. Interestingly, unlike molecules that exclusively inhibit all of TRPV1’s modalities, in vivo studies show that APHC1 and APHC3 do not cause hyperthermia [80]. Rather, these molecules decrease core body temperatures commonly observed during administration of TRPV1 selective agonists [80]. In accordance with their different inhibitory mechanisms, injection of APHC1 induced a sharp fall in body temperature of −0.8 °C within 30 min after administration, whereas APHC3 produced a slow decrease of −0.6 °C reached 60 min [80].

These toxins give an interesting insight into the possibility of TRPV1 modulation through an intermediate state. A potential aim for the development of novel analgesics through TRPV1 may be to avoid inhibiting TRPV1 as a whole and instead target a specific modality. These toxins provide an indication of the possible effects of partially inhibiting TRPV1.

### 6.4. Structural Features

The three highly homologous toxins have been characterized, differing in four of their 56 residues, and are estimated to be approximately 6187.0 Da each [78]. They contain unique features of protease inhibitors derived from bovine pancreas, such as a disulfide rich α/β structure common to BPTI/Kunitz-type enzymes [78]. Other molecules common to this group include SHPI-1, a trypsin inhibitor (85% homology with APHC), KAL-1, a potassium channel blocker, and calcicludine, a calcium channel blocker (52% APHC homology) [78].

Molecular modeling of the toxin on the background of cryo-EM structures of TRPV1 suggest that functionally important residues for toxin interactions are in the outer pore domain, involving the pore helix and two extracellular loops, and overlapping with the proton binding site [79]. Considering this, antagonism by the toxin at acidic pH may involve competitive inhibition rather than allosteric, in the case of other modalities [79]. Arginine residues in the toxin’s C terminal mediate the strongest interactions between the toxin and TRPV1 outer pore domain [79]. Specifically, R48 of the toxin interacts with E648, E651, and Y653 (rTRPV1) of TRPV1’s outer pore domain [79]. Similar to previously described toxins, APHC1 interacts simultaneously with two TRPV1 subunits as R51 of APHC1 interacts with E636 and Y627, and R55 of APHC1 interacts strongly with D646 of the adjacent subunit [79]. Moreover, two conformational states of the toxin, which depend on an open or closed TRPV1 channel, suggest that there is a two-way effect of conformational rearrangements of the channel affecting the toxin and the toxin affecting the channel [79]. Specifically, in the open state, the pore helix of TRPV1 pushes APHC1 about 2.5 Å in the external direction [79]. In this way, APHC toxins stabilize an intermediate state, producing a bimodal effect on TRPV1 gating [79].

## 7. *Heteractis crispa* RG 21 (HCRG21)

### 7.1. Introduction

Toxins with a kunitz-type domain act as protease inhibitors, yet some of these peptides have an additional neurotoxic activity as they also inhibit ion channels [81]. Such dual activity is observed in HCRG21, a kunitz-type peptide from the venom of *Heteractis crispa* [34]. This sea anemone toxin inhibits both trypsin and TRPV1 [34]. The structure of this bifunctional toxin contains three disulfide bonds and has a molecular mass of 6228 Da [34]. The HCRG21 sequence is highly homologous (82%–95%) to APHC1-3, TRPV1 inhibiting toxins from the same venom described above [34,78]. In addition to TRPV1 inhibitors, HCRG21 also shares high identity (84%) with ShPI-1, a potent inhibitor of several voltage-gated potassium channels [34]. However, HCGR21 does not modulate the activity of these channels [34].

### 7.2. Functional Aspects

TRPV1 inhibition by HCRG21 is dose-dependent with an IC_50_ = 6.9 ± 0.4 µM when co-applied with 1 µM capsaicin [34]. The unclear evolutionary benefit of inhibiting TRPV1 combined with its low potency suggests that this toxin targets additional ion channels. Furthermore, it is not clear whether TRPV1 is inhibited by physiologically relevant toxin concentrations. However, unlike APHC1, HCRG21 is a full channel antagonist [34].

### 7.3. Effect on Nociception

Introduction of TRPV1 antagonists to the clinic as new analgesics was hampered as these inhibitors produced serious, adverse effects, namely hyperthermia and insensitivity to scalding heat [82,83,84]. Although these are on-target effects, it is thought that inhibiting one of the modalities activating TRPV1 would produce the desired analgesic effect while maintaining a favorable safety profile. However, HCRG21’s ability to block TRPV1 activation by modalities other than capsaicin is yet to be reported.

### 7.4. Structural Features

Most residues that were found to be important in APHC1 binding to TRPV1 (E6, T14, E38, R48, and R51) are conserved in HCRG21, except for V31, which is substituted by proline [34]. This substitution could underlie the difference in efficacy between these two toxins. However, the significance of the aforementioned residues in HCRG21 activity is yet to be confirmed. Using homology modeling and molecular dynamics simulations, HCRG21 was hypothesized to interact with the regulatory domain in the intracellular TRPV1 C-terminal where PIP2 also binds the channel [34]. Nonetheless, both the ability of HCRG21 to penetrate the membrane and to block capsaicin currents through this site were not tested. A far likelier possibility, which was also suggested using computational analysis, is that HCRG21 binds the outer vestibule sitting directly on the TRPV1 channel pore thus presumably blocking the conductance of ions [34]. Conformational changes in the outer pore region and interaction with a residue in the channel’s selectivity filter were also predicted upon toxin binding [34]. However, this computational data is not yet backed by any functional analysis experiments.

## 8. Toxins and the TRPV1 Outer Pore Domain

**Figure 5:**
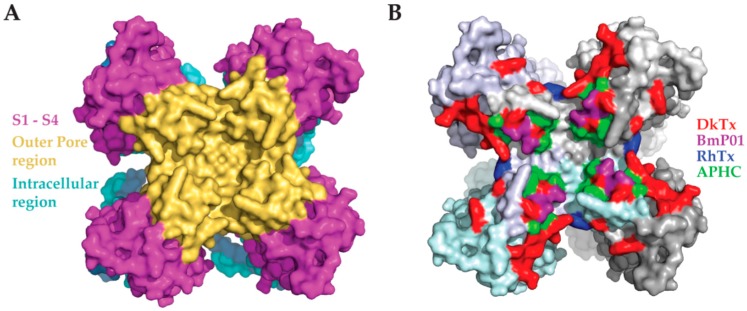
Significant amino acids for toxin-induced modulation of TRPV1 activity. (**A**) The outer pore region is collectively colored in gold, and S1–S4 are labeled in fuchsia. Intracellular structures are blue. PDB ID: 5IRZ. (**B**) Key amino acids indicated in channel activation by each individual toxin are labeled according to the toxin: DkTx, Red; RhTx, Blue; BmP01, Purple; APHC, Green. Whole subunits are lightly colored to visualize the interface between adjacent subunits. PDB ID: 5IRZ.

Toxins have evolved over thousands of years to target physiologically significant processes in order to exert a robust and acute effect in their victims [6]. To do so, toxins may modulate the activity of central ion channels by binding or affecting functionally important domains in these proteins [6,85]. Thus, toxins have been instrumental in understanding the structure and function of ion channels so far [41]. Common ion channel domains targeted by toxins include the voltage sensor, ion permeable pathway, and agonist binding sites [6]. Another channel site affected by toxins is the outer pore domain [6]. By binding to this region some toxins, like charybdotoxin, serve as a cork occluding ion conductance through the pore and directly inhibit channel activity [86]. However, other toxins either inhibit or activate channels by binding to sites in the outer pore domain that are involved in the channel’s gating mechanism. Accordingly, as reviewed here, several toxins were shown to activate, modulate, or inhibit TRPV1 by binding to the outer pore domain of the channel (Figure 5 and Table 1) [34,36,51,61]. These findings signify the outer pore region of TRPV1 as a common domain for binding and gating of the channel by different peptide animal toxins, and highlight the importance of the outer pore domain in TRPV1 activation.

The outer pore region also plays a pivotal role in TRPV1 activation evoked by various stimuli other than toxins. For instance, extracellular protons potentiate and activate TRPV1 through glutamate residues (E600 and E648, respectively) found in this region [5,87]. Furthermore, protons were also found to perturb outer pore structure leading to sub-conductance TRPV1 currents [88]. Divalent cations were suggested to induce substantial conformational rearrangements in the outer pore region, which potentiates channel activity [89]. In addition, mutagenesis experiments revealed gain-of-function mutations within the extracellular pore domain [90]. Residues in this region were also found to be essential for heat activation of TRPV1 [91,92]. However, this does not necessarily mean that the channel’s heat sensor lies within the outer pore domain, as other protein domains were also found to be equally involved in this process [93,94,95].

The proximity of so many binding sites and functionally important domains in the outer pore region raises the possibility that some toxins allosterically modulate TRPV1 activation by other stimuli. Indeed, RhTx was shown to reduce the threshold for heat activation by 6 °C [51]. In addition, BmP01 was found to affect the protons-induced gating machinery. The preferential binding of DkTx to the open TRPV1 conformation suggests that this toxin may also enhance the channel’s response to other modalities in physiological settings [36,61].

Structural analyses of the TRPV1 channel in distinct conformations have confirmed that the outer pore domain undergoes substantial structural reorganization, which is associated with a shift in the pore helix relative position during the gating process [11,96]. In contrast, the S1–S4 domain remains static in different channel conformations and was suggested to serve as a scaffold for the S5-Pore-S6 domain [56]. TRPV1 and voltage gated ion channels share a similar topology and three-dimensional structure [46,97]. In voltage-gated ion channels, the outer pore region was shown to be relatively stationary during transitions between the apo and open states, while the S1–S4 domain where the voltage sensor is situated is highly dynamic [10,98]. Reflecting the differential gating mechanisms in these two channel types, VaTx3 inhibits Kv channels by binding to the channel’s voltage sensor region (S3–S4) yet activates TRPV1 via the outer-pore domain to cause net hyper-excitability in effected neurons [32,36].

## 9. Conclusions

In the past five years, many advances have been made in our understanding of TRPV1 activation, structure, and potential roles in pain management therapy, greatly due to the rich collection of animal toxins that target this ion channel [6,11,41]. In this review, we discuss the molecular traits of these toxins that have been pivotal to our understanding of TRPV1, with the aims of elucidating the detailed functioning of the pain system. It is only in this way that we can develop stronger, more precise tools to manipulate and control the nociceptive system.

Considering the discussed toxins, we find remarkable diversity in terms of structure, functional mechanism, and effect. However, the outer pore region of TRPV1 serves as a common and complex site in which toxins and other modalities (i.e., heat and protons) converge to modulate TRPV1 gating in distinct manners. For example, DkTx’s unique structure produces a strong TRPV1 response, but does so through slow, prolonged TRPV1 activation by binding to the channel’s open conformation [36]. In contrast, aversive behavior is also seen in RhTx, which produces strong, capsaicin-like TRPV1 activation [51]. Through RhTx, we also understand that conformational changes due to the ambient temperature may also affect the binding of the molecule [51]. Similarly, TRPV1 activation by BmP01 was shown to be potentiated by low pH values [59]. Thus, these toxins may take advantage of existing conditions at the site of injected venom (the presence of inflammatory mediators, protons, and body temperature) as a general strategy to enhance their potency and effect. Moreover, considering the recently characterized *Echis coloratus* activity on TRPV1, we can understand that, although all toxins thus far target the outer pore domain, key amino acids in TRPV1 activation remain to be described [69]. Other toxins, such as the APHC family or HCRG21 which display analgesic properties, have an unclear purpose for inhibiting TRPV1 [34,78]. Nonetheless, these toxins offer insight into the behavioral effects of inhibiting TRPV1.

## Figures and Tables

**Table 1:** Key toxin features and interactions with TRPV1.

Species	Toxin		
**Chinese earth tiger tarantula** ***Chilobrachys guangxiensis***	Double-knot toxin (DkTx) [36]	**Key Amino Acids**	*TRPV1*: Y453, R534, K535, E536, I599, S629, Y631, F649, T650, A657, N652, D654, F655, K656, A657, V658, F659 *DkTx*: K1: W11, G12, K14, and F27 K2: W53, G54, K56 and F67
		**Behavioral effects**	Unknown
		**Potency**	EC_50_ = 0.23 µM
**Chinese red-headed centipede** ***Scolopendra subspinipes mutilans***	RhTx [51]	**Key Amino Acids**	*TRPV1:* D602, Y632, T634, Possibly L461 *RhTx*: D20, K21, Q22, R15, E27
		**Behavioral effects**	Acute pain response when injected into mice
		**Potency**	EC_50_ = 521.5 ± 162.1 nM
**Chinese Scorpion** ***Mesobuthus martensii***	BmP01 [59]	**Key Amino Acids**	*TRPV1:* E648, T651, E652
		**Behavioral effects**	Injection of 500 µM BmP01 evokes a pain response in wt mice but not in TRPV1 KO mice
		**Potency**	EC_50(Ph=6.5)_ = 3.76 ± 0.4 µM EC_50(Ph=7.5)_ = 169.5 ± 12.3 µM
**Palestine saw-scaled viper** ***Echis coloratus***	F13 [69]	**Key Amino Acids**	Unknown
		**Behavioral effects**	Unknown
		**Potency**	Unknown
**Sebae anemone** ***Heteractis crispa***	Heteractis crispa RG 21 (HCRG21) [34]	**Key Amino Acids**	*HCRG21*: E6, T14, P31 E38, R48, R51
		**Behavioral effects**	Unknown
		**Potency**	IC_50_ = 6.9 ± 0.4 µM
	Analgesic polypeptide Heteractis crispa (APHC1-3) [78]	**Key Amino Acids**	*TRPV1:* D648, E651, Y653, E636, Y627, D646 *APHC*: V31, R48, R51, R55
		**Behavioral effects**	▪ dose-dependent inhibition of thermal nociception ▪ APHC1 decreases both phases of the formalin test (acute and inflammatory pain). APHC3 attenuates only inflammatory phase ▪ attenuates thermal hyperalgesia observed during CFA injection ▪ Injection of APHC1 decreased body temperature by −0.8 °C within 30 min after administration. Injection of APHC3 decreases body temperature by 0.6 °C 60 min after administration.
		**Potency**	APHC1: IC_50_ = 6.9 ± 0.4 µM APHC3: IC_50_ = 18 nM
